# Radial Basis Function Neural Network Application to Power System Restoration Studies

**DOI:** 10.1155/2012/654895

**Published:** 2012-06-26

**Authors:** Iman Sadeghkhani, Abbas Ketabi, Rene Feuillet

**Affiliations:** ^1^Department of Electrical Engineering, Najafabad Branch, Islamic Azad University, Najafabad 8514143131, Iran; ^2^Department of Electrical Engineering, University of Kashan, Kashan 8731751167, Iran; ^3^Grenoble Electrical Engineering Lab (G2ELab), Grenoble INP, BP46, 38402 Saint Martin d'Hères, Cedex, France

## Abstract

One of the most important issues in power system restoration is overvoltages caused by transformer switching. These overvoltages might damage some equipment and delay power system restoration. This paper presents a radial basis function neural network (RBFNN) to study transformer switching overvoltages. To achieve good generalization capability for developed RBFNN, equivalent parameters of the network are added to RBFNN inputs. The developed RBFNN is trained with the worst-case scenario of switching angle and remanent flux and tested for typical cases. The simulated results for a partial of 39-bus New England test system show that the proposed technique can estimate the peak values and duration of switching overvoltages with good accuracy.

## 1. Introduction

In recent years, due to economic competition and deregulation, power systems are being operated closer and closer to their limits. At the same time, power systems have increased in size and complexity. Both factors increase the risk of major power outages. After a blackout, power needs to be restored as quickly and reliably as possible and, consequently, detailed restoration plans are necessary [1, 2]. If the frequency characteristic of the system shows resonance conditions around multiples of the fundamental frequency, very high and weakly damped temporary overvoltages (TOVs) of long duration may occur when the system is excited by a harmonic disturbance, such as the switching of lightly loaded transformers or transformer saturation [1, 3–5].

Overvoltages can be classified as transient overvoltages, sustained overvoltages, harmonic resonance overvoltages, and overvoltages resulting from Ferroresonance. Excessive sustained overvoltages may lead to damage of transformers and other power system equipment. Transient overvoltages are a consequence of switching operations on long transmission lines, or the switching of capacitive devices, and may result in arrester failures. Ferroresonance is a nonharmonic resonance characterized by overvoltages whose waveforms are highly distorted and can cause catastrophic equipment damages [1]. Overvoltage will put the transformer into saturation, causing core heating and copious harmonic current generation. Circuit breaker called upon to operate during periods of high voltage will have reduced interrupting capability. At some voltage even the ability to interrupt line-charging current will be lost [1, 6–8].

**Figure 1:**
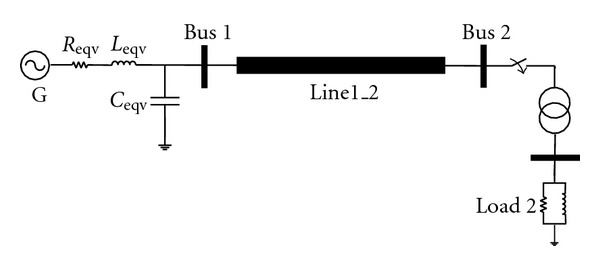
Sample system for energizing a transformer.

In [9], overvoltages caused by transformer energization have been evaluated using radial basis function neural network (RBFNN). Also in [10], switching overvoltages peak have been estimated using multilayer perceptron neural network (MLP-ANN) during transmission line energization. But developed ANNs in both works are applicable just for trained system. In this paper, by using equivalent parameters as ANN inputs during training process, developed ANN is applicable to every studied system and trained only once for a sample system (Figure 1). In [9], remanent flux is an ANN input, but measurement of this parameter is very difficult. In this paper, proposed index is developed to determine worst case of switching condition and remanent flux; thus, there is no requirement to measure and determine remanent flux and switching angle. This issue is described in Section 5. 

A MATLAB/Simulink-based simulation tool [11, 12], power system blockset (PSB), is used for computation of temporary overvoltages. In order to study temporary overvoltages for a large number of possible system configurations, it is necessary to run many time-domain simulations resulting in a large amount of simulation time. A way to limit the overall calculation time is to reduce the number of simulations by applying analytical or knowledge-based rules to discard a number of system configurations before an actual time-domain simulation is carried out. This paper presents the artificial neural network (ANN) application for estimation of peak and duration overvoltages under switching transients during transformer energization. The proposed approach can give the maximum switching overvoltage and its duration which may be helpful to the operator during system restoration. Also it can be used as training tool for the operators. The proposed ANN is expected to learn many scenarios of operation. To give the maximum peak overvoltage and it's duration in a shortest computational time which is the requirement during online operation of power systems. In the proposed ANN we have considered the most important aspects, which influence the transient overvoltages such as voltage at transformer bus before switching, equivalent resistance, equivalent inductance, equivalent capacitance, line length, switching angle, saturation curve slope, and remanent flux. This information will help the operator to select the proper sequence of transformer to be energized safely with transients appearing safe within the limits. Results of the studies are presented for a partial of 39-bus New England test system to illustrate the proposed approach.

## 2. Study System Modelling

### 2.1. PSB

Simulations presented in this paper are performed using the PSB [11]. The simulation tool has been developed using state variable approach and runs in the MATLAB/Simulink environment. This program has been compared with other popular simulation packages (EMTP and Pspice) in [12]. The user friendly graphical interfaces of PSB enable faster development for power system transient analysis.

### 2.2. Generator Model

In [13] generators have been modeled by generalized Park's model that both electrical and mechanical part are thoroughly modeled, but it has been shown that a simple static generator model containing an ideal voltage source behind the subtransient inductance in series with the armature winding resistance can be as accurate as the Park model. Thus in this work, generators are represented by the static generator model. Phases of voltage sources are determined by the load flow results. 

### 2.3. Transmission-Line Model

Transmission lines are described by the distributed line model. This model is accurate enough for frequency dependent parameters, because the positive sequence resistance and inductance are fairly constant up to approximately 1 KHz [14] which cover the frequency range of harmonic overvoltages phenomena. 

### 2.4. Transformer Model

The model takes into account the winding resistances (*R*_1_, *R*_2_), the leakage inductances (*L*_1_, *L*_2_), and the magnetizing characteristics of the core, which is modeled by a resistance, *R*_*m*_, simulating the core active losses and a saturable inductance, *L*_sat⁡_. The saturation characteristic is specified as a piecewise linear characteristic [15].

### 2.5. Load and Shunt Devices Model

All of the loads and shunt devices, such as capacitors and reactors, are modeled as constant impedances.

## 3. Study for Temporary Overvoltages during Restoration

The estimation of harmonic overvoltages is important during system restoration. These are a result of network resonance frequencies close to multiples of the fundamental frequency. They can be excited by harmonic sources such as saturated transformers, power electronics, and so forth. They may lead to long-lasting overvoltages resulting in arrester failures and system faults [2].

The major cause of harmonic resonance overvoltage problems is the switching of lightly loaded transformers at the end of transmission lines. The harmonic-current components of the same frequency as the system resonance frequencies are amplified in case of parallel resonance, thereby creating higher voltages at the transformer terminals. This leads to a higher level of saturation, resulting in higher harmonic components of the inrush current that again results in increased voltages. This can happen particularly in lightly damped systems, common at the beginning of a restoration procedure when a path from a black-start source to a large power plant is being established and only a few loads are restored yet [1, 16].

**Figure 2:**
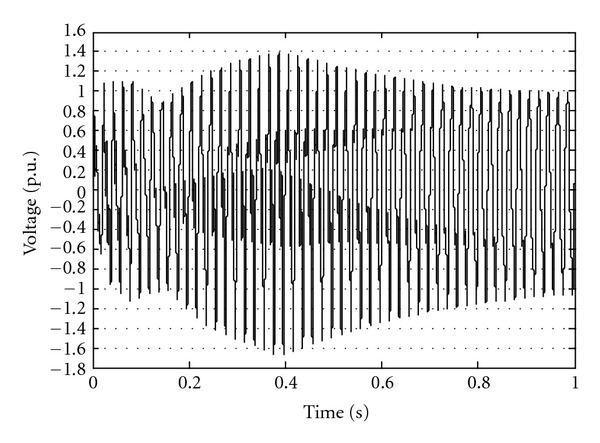
Voltage at bus 2 after switching of transformer.

The sample system considered for explanation of the proposed methodology and training ANN is a 400 kV extra high voltage (EHV) network shown in Figure 1. The normal peak value of any phase voltage is 400 $\sqrt{2}/\sqrt{3}$ kV and this value is taken as base for voltage p.u. In the system studies 100 MVA as a base power is considered.  Figure 2 shows a sample switching overvoltages at bus 2 when transformer is energized.

In practical system a number of factors affect the overvoltages factors due to energization or reclosing. In this paper following parameters is considered.

- Voltage at transformer bus before switching.
- Equivalent resistance of the network.
- Equivalent inductance of the network.
- Equivalent capacitance of the network.
- Line length.
- Closing time of the circuit breaker poles.
- Saturation curve slope.
- Remanent flux.

The equivalent circuit parameters are added to ANN inputs to achieve good generalization capability for trained ANN. In fact, in this approach ANN is trained just once for sample system of Figure 1. Since ANN training is based on equivalent circuit parameters, developed ANN can be used for every studied system. Also, to reduce time-domain simulations, a new approach based on the worst case condition determination is proposed in Section 5. 

**Figure 3:**
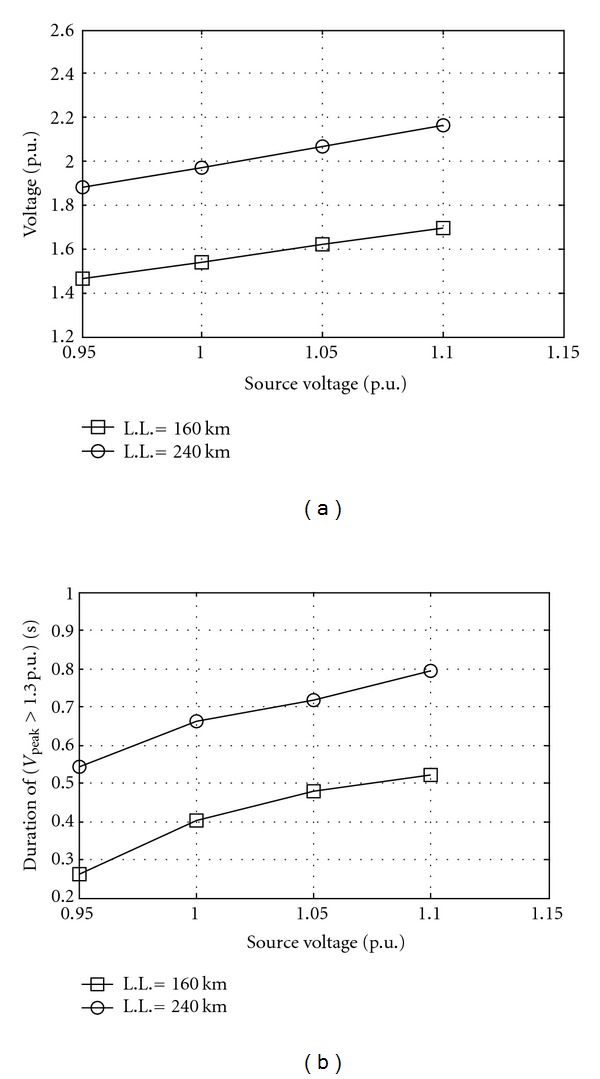
Overvoltage at bus 2 as source voltage while equivalent resistance 0.003 p.u., equivalent inductance 0.025 p.u., equivalent capacitance 1.2825 p.u., switching angle 45°, saturation curve slope 0.32 p.u., and remanent flux 0.8 p.u. L.L. is line length. (a) Peak and (b) duration.

**Figure 4:**
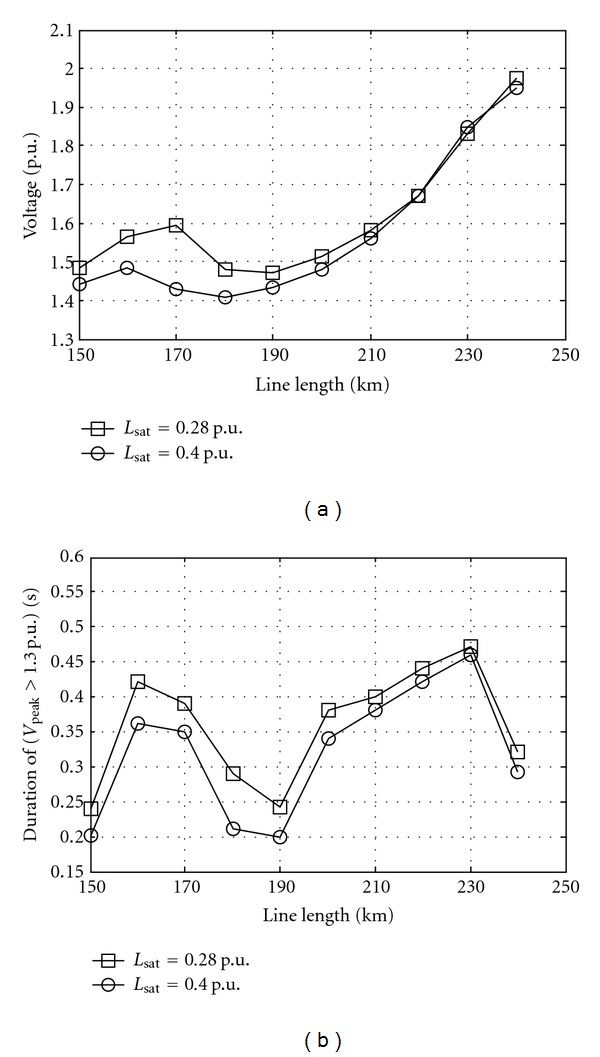
Overvoltage at bus 2 as line length while source voltage 1 p.u., equivalent resistance 0.003 p.u., equivalent inductance 0.025 p.u., equivalent capacitance 1.282 p.u., switching angle 45°, and remanent flux 0.8 p.u. *L*_sat⁡_ is saturation curve slope. (a) Peak and (b) duration.

**Figure 5:**
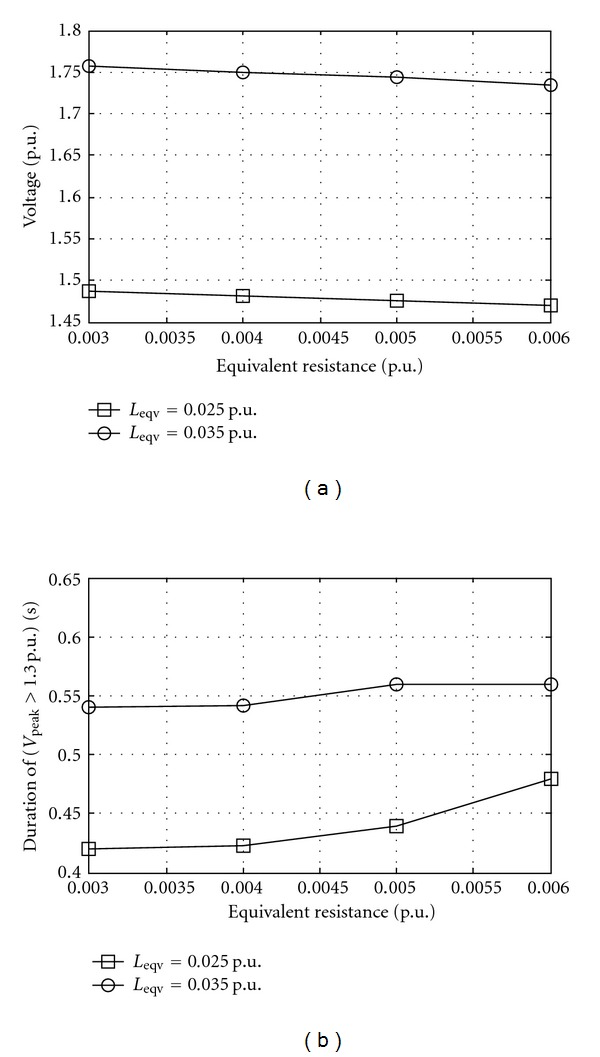
Overvoltage at bus 2 as equivalent resistance while source voltage 1 p.u., equivalent capacitance 1.8912 p.u., line length 200 km, switching angle 30°, saturation curve slope 0.32 p.u., and remanent flux 0.6 p.u. *L*_eqv_ is equivalent inductance. (a) Peak and (b) duration.

**Figure 6:**
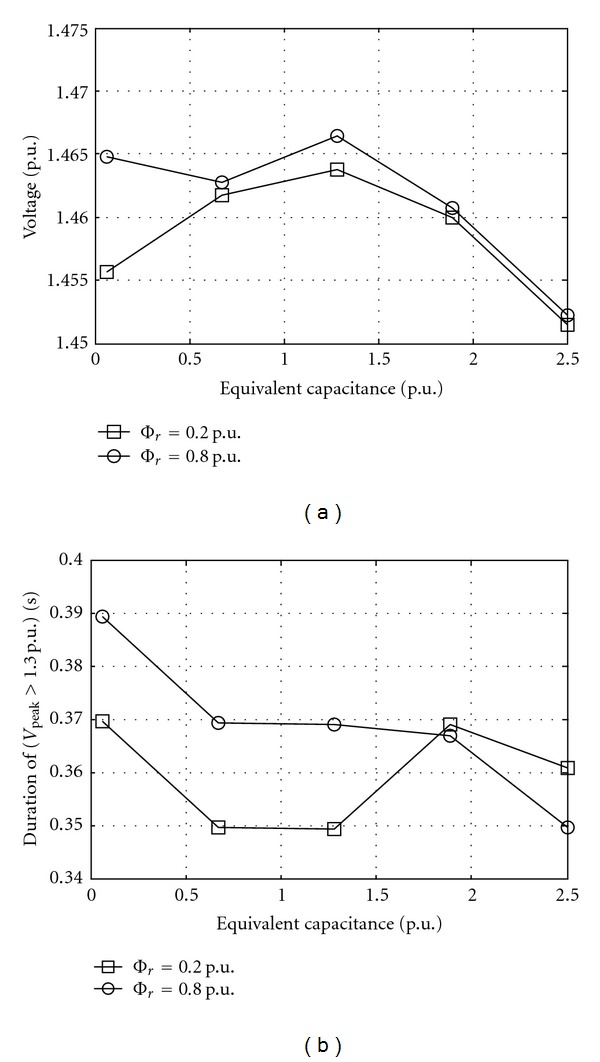
Overvoltage at bus 2 as equivalent capacitance while source voltage 1 p.u., equivalent resistance 0.003 p.u., equivalent inductance 0.02 p.u., line length 200 km, switching angle 30°, and saturation curve slope 0.32 p.u. Φ_r_ is remanent flux. (a) Peak and (b) duration.

Source voltage affects the overvoltage strongly.  Figure 3 shows the effect of source voltage on overvoltage at different line lengths.  Figure 4 shows the effect of line length on overvoltages at different saturation curve slope. The saturation curve, and especially the *L*_sat⁡_, that is, the final slope of this curve, is a key point for the computation of the inrush currents. The transformer manufacturer provides a *L*_sat⁡_ slope value with a dispersion usually considered of ±20%.  Figure 5 shows effect of equivalent resistance on overvoltages at different equivalent inductance.  Figure 6 shows the effect of equivalent capacitance on overvoltages at different remanent flux.

## 4. The Radial Basis Function Neural Network

**Figure 7:**
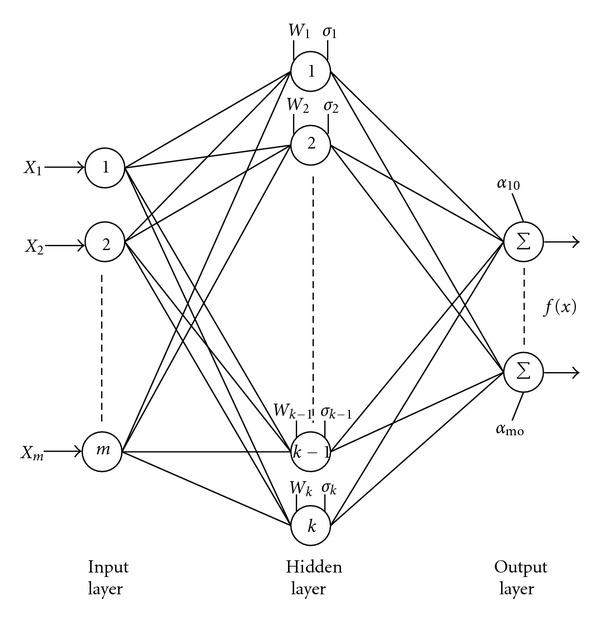
The structure of RBF neural network.

Figure 7 shows the structure of the RBF neural network, which comprises of three layers. The hidden layer possesses an array of neurons, referred to as the computing units. The number of such units can be varied depending on user's requirement [17, 18]. Different basis functions like spline, multiquadratic, and Gaussian functions have been studied, but the most widely used one is the Gaussian type. In comparison to the other types of neural network used for pattern classification like back propagation feedforward networks, the RBF network requires less computation time for learning and has a more compact topology. The Gaussian RBF is found not only suitable in generalizing a global mapping but also in refining local features without altering the already learned mapping. Each hidden unit in the network has two parameters called a center ( *ω* ) and a width ( *σ* ) associated with it. The response of one such hidden unit to the network input **X**, **X** = [*x*_1_,*x*_2_,…,*x*_**n**_]^*T*^ is expressed as:

(1)ϕk(X)=exp⁡(−1σk2||X−ωk||2),

where *ω*_*k*_  is the center vector for *k*th hidden unit, **σ*k *is the width of the Gaussian function, and |||| denotes the Euclidean norm. The output layer comprises a number of nodes depending on the number of fault types to be classified which perform simple summation. The response of each hidden unit (1) is scaled by its connecting weights ( *α* 's) to the output nodes and then summed to produce the overall network output. The overall network output is expressed as:

(2)fm(X)=αmo+∑k=1Nαmkϕk(X),

where *N* indicates the total number of hidden neurons in the network, *α*_mk_ is the connecting weight of the *k*th hidden unit to *m*th output node, and *α*_mo_ is the bias term for the corresponding *m*th output neuron. The learning process of the RBFNN involves with the allocation of new hidden units and tuning of network parameters. The learning process is terminated when the output error goes under the defined threshold [19].

## 5. Proposed Method for Harmonic Overvoltages Study

### 5.1. Worst-Case Condition Determination for Overvoltages Simulation

Normally for harmonic overvoltages analysis, the worst case of the switching angle and remanent flux must be considered which it is a function of switching time, transformer characteristics and its initial flux condition, and impedance characteristics of the switching bus [15]. Using the worst switching angle and remanent flux, the number of simulations for each case can be reduced significantly.

In order to determine worst-case switching time and remanent flux, the following index is defined as:

(3)W=∑h=210Zjj(h)·Ij(h,t0,ϕr),

where *t*_0_ is the switching time, *ϕ*_*r*_ is initial transformer flux, and *h* is harmonic order. This index can be a definition for the worst-case switching condition and remanent flux. Using a numerical algorithm, one can find the switching time and remanent flux for which *W* is maximal (i.e., harmonic overvoltages is maximal). 

**Figure 8:**
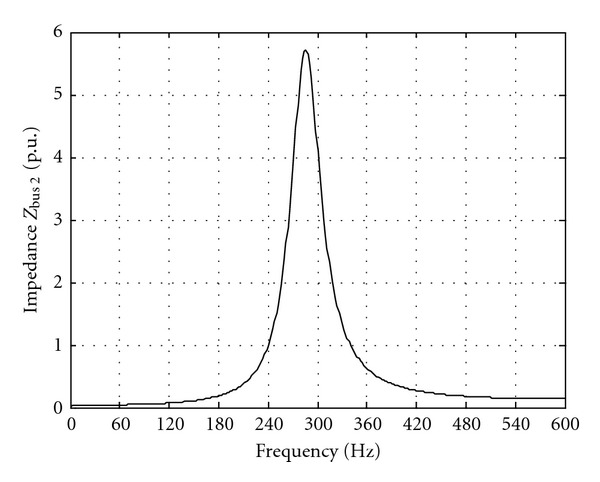
Impedance at bus 2.

**Figure 9:**
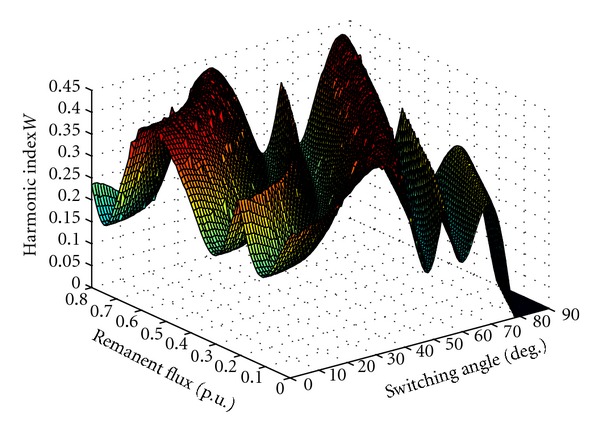
Changes of *W* index with respect to current starting angle and remanent flux.

**Figure 10:**
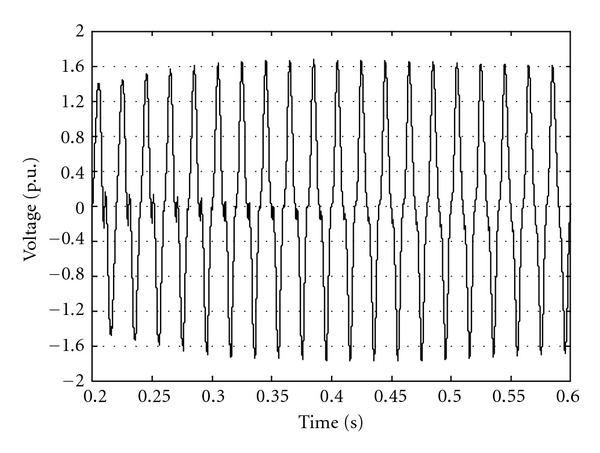
Voltage at bus 2 after switching of transformer for worst-case condition.

Figure 8 shows the result of the PSB frequency analysis at bus 2. The magnitude of the thevenin impedance, seen from bus 2, Zbus2 shows a parallel resonance peak at 286 Hz.  Figure 9 shows changes of *W* index with respect to the current starting angle and remanent flux.  Figure 10 shows voltage at bus 2 after transformer switching for the worst-case condition (i.e., switching angle 78° and remanent flux 0.64 p.u.). For temporary overvoltages, the overvoltage duration has to be taken into account in addition to the amplitude [16].  Table 1 summarizes the results of overvoltages simulation for four different switching angle and remanent flux that verify the effectiveness of *W* index.

### 5.2. Steps of Assessment and Estimation of Harmonic Overvoltages

The steps for harmonic overvoltages assessment and estimation follow.

1. Determine the characteristics of transformer that should be energized.
2. Calculate the *Z*_*ii*_(*h*) at the transformer bus for *h* = 2*f*_0_,…, 10*f*_0_.
3. Compute the worst switching angle and remanent flux for simulation.
4. Run PSB simulation.
5. Determine overvoltage peak and duration.
6. Repeat the steps 1 to 5 with various system parameters to learn artificial neural network.
7. Test artificial neural network with different system parameters.

### 5.3. Training Artificial Neural Network

All experiments have been repeated for different system parameters (2000 sets). For producing these sets, ANN input, that is, source voltage (voltage at transformer bus before switching), equivalent resistance, equivalent inductance, equivalent capacitance, line length, and saturation curve slope have been changed in steps of 0.05 p.u., 0.001 p.u., 0.01 p.u., 0.304 p.u., 20 km, and 0.04 p.u., respectively. 1000 sets were used to train RBFNN and 1000 sets were used to test RBFNN. RBFANN learned in 62 epochs. After learning, all parameters of the trained networks have been frozen and then used in the retrieval mode for testing the capabilities of the system on the data not used in learning. The testing data samples have been generated through the PSB program by placing the parameter values not used in learning, by applying different parameters. A large number of testing data have been used to check the proposed solution in the most objective way at practically all possible parameters variation. Percentage error is calculated as:

(4)error(%)=|OANN−OPSB|OPSB×100,

where *O*_ANN_ is output (overvoltages peak and duration) calculated by ANN, and *O*_PSB_ refers to overvoltages peak and duration calculated by PSB. 

**Figure 11:**
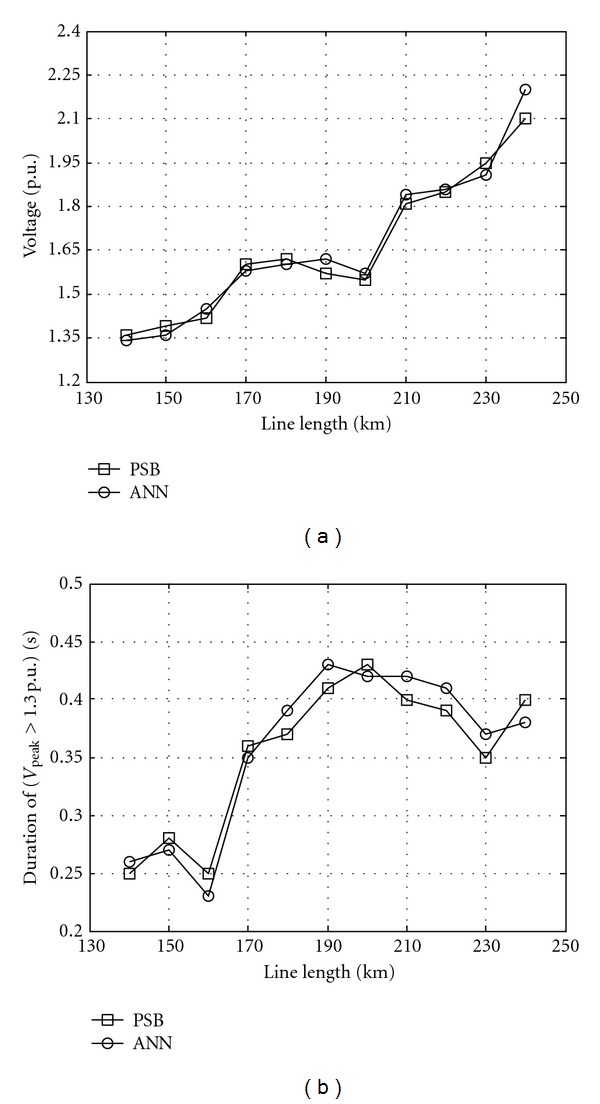
Overvoltages versus line length at bus 2 simulated by ANN and PSB while source voltage 1 p.u., equivalent resistance 0.004 p.u., equivalent inductance 0.03 p.u., equivalent capacitance 1.282 p.u., switching angle 15°, remanent flux 0.5 p.u., and saturation curve slope 0.32 p.u. (a) Peak and (b) duration.

**Figure 12:**
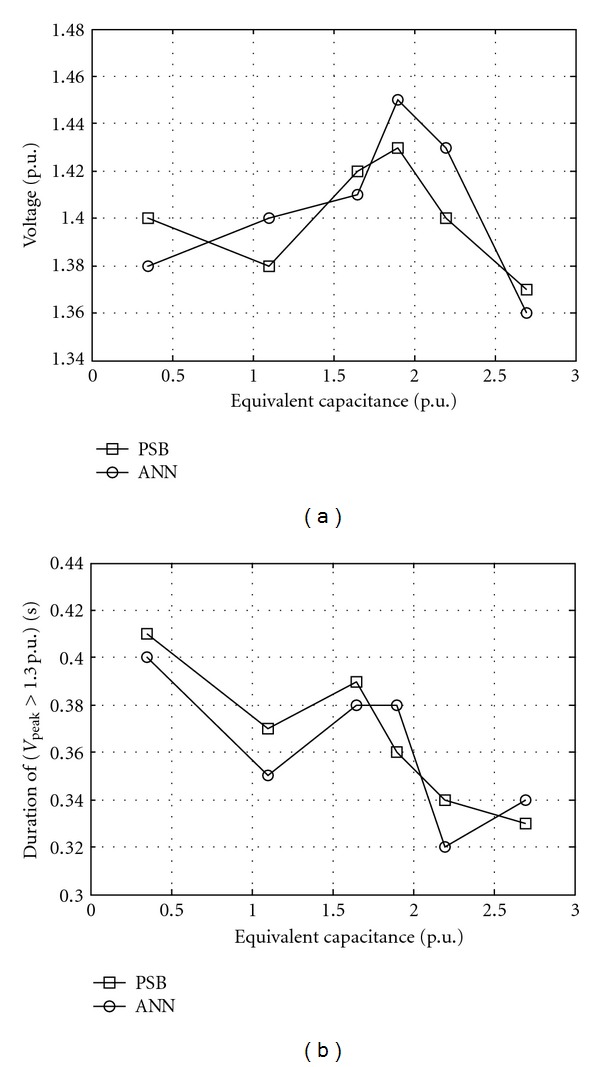
Overvoltages versus equivalent capacitance at bus 2 simulated by ANN and PSB while source voltage 1 p.u., equivalent resistance 0.003 p.u., equivalent inductance 0.03 p.u., line length 180 km, switching angle 45°, saturation curve slope 0.32 p.u., and remanent flux 0.2 p.u. (a) Peak, (b) Duration.

Results for a sample test data are presented in Table 2 and Figures 11 and 12. Values in column *V*_PSB_ are the absolute values of peak voltage at bus 2 calculated by PSB program in p.u. where the *V*_RBFNN_ values are the values simulated by trained neural network. Also Values in column *T*_PSB_ are the values of overvoltage duration calculated by PSB program in seconds and *T*_RBFNN_ values are the values simulated by trained neural network.  Figure 11 shows overvoltages peak and duration at bus 2 against the line length and Figure 12 shows overvoltage peak and duration at bus 2 against the equivalent capacitance.

In the next section, the proposed model tested with portion of 39-bus New England test system. Various cases of transformer energization are taken into account and corresponding peak and duration values are estimated from trained model.

## 6. Case Study

In this section, the proposed algorithm is demonstrated for two case studies that are a portion of 39-bus New England test system, of which its parameters are listed in [20]. The simulations are undertaken on a single-phase representation.

Using the equivalent circuit parameters the artificial neural network is trained for system of Figure 1. Thus studied systems should be converted to equivalent circuit of Figure 1, where values of equivalent resistance, inductance, and capacitance are calculated. These values are used in trained ANN to estimate overvoltages peak and duration.

### 6.1. Case 1

**Figure 13:**
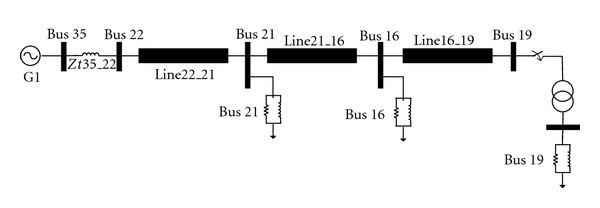
Studied system for case 1.

Figure 13 shows a one-line diagram of a portion of 39-bus New England test system which is in restorative state. The generator at bus 35 is a black-start unit. The load 19 shows cranking power of the later generator that must be restored by the transformer of bus 19. When the transformer is energized, harmonic overvoltages can be produced because the transformer is lightly loaded. The equivalent circuit of this system that seen behind bus 16 is determined and values of equivalent resistance, equivalent inductance, and equivalent capacitance are calculated. In other words, the case study system is converted to equivalent system of Figure 1. Values of equivalent resistance, equivalent inductance and equivalent capacitance are 0.00291 p.u., 0.02427, and 2.474 p.u., respectively. For testing trained ANN, values of voltage at transformer bus (bus 19) and line length are varied and overvoltage peak and duration are calculated from trained ANN.  Table 3 contains the some sample result of test data of case 1.

### 6.2. Case 2

**Figure 14:**
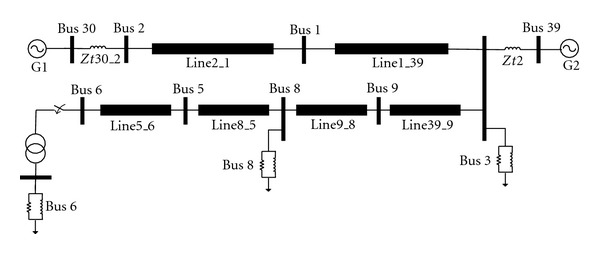
Studied system for case 2.

As another example, the system in Figure 14 is examined. In the next step of the restoration, unit at bus 6 must be restarted. In order to provide cranking power for this unit, the transformer at bus 6 should be energized. In this condition, harmonic overvoltages can be produced because the load of the transformer is small.

After converting this system to equivalent circuit of Figure 1 and calculating equivalent circuit seen from bus 5, various cases of transformer energization are taken into account and corresponding peak and duration overvoltages are computed from PSB program for system of Figure 14 and trained ANN. In this case, values of equivalent resistance, equivalent inductance and equivalent capacitance are 0.00577 p.u., 0.02069, and 0.99 p.u., respectively. Summary of few results are presented in Table 4. It can be seen from the results that the ANN is able to learn the pattern and give results to acceptable accuracy.

## 7. Conclusion

This paper presents new approach to study transformer switching overvoltages during power system restoration. For this purpose, an RBFNN has been used to estimate the peak and duration overvoltages due to transformer *‎*energization. Equivalent circuit parameters have been used as RBFNN inputs to achieve good generalization capability for trained ANN. The results from this scheme are close to results from the conventional method and can assists prediction of the overvoltage of other case studies within the range of the training set. The proposed ANN approach is tested on a partial 39-bus New England test system. Since the proposed method is performed based on the worst case switching angle and remanent flux, it omits time-consuming time-domain simulations and is therefore suitable for real-time applications during system restoration. Also it can be used as a training tool for the operators.

## Figures and Tables

**Table 1:** Effect of switching time and remanent flux on the maximum of overvoltages and duration of *V*_peak_ > 1.3 p.u.

Switching Angle (deg.)	Remanent Flux (p.u.)	*V*_peak_ (p.u.)	Duration of (*V*_peak_ > 1.3 p.u.) (s)
78	0.64	1.7739	0.6027
78	0.25	1.5416	0.4976
45	0.64	1.5984	0.5482
27	0.31	1.6501	0.5879

**Table 2:** Some sample testing data and output.

*V*	*R*_eqv_	*L*_eqv_	*C*_eqv_	L. L.	*L*_sat⁡_	*V*_PSB_	*V*_RBFNN_	Error_*V*_	*T*_PSB_	*T*_RBFNN_	Error_*T*_
0.951	0.003	0.025	1.2825	165	0.3	1.5338	1.5534	1.2749	0.4204	0.4328	2.9517
0.995	0.003	0.025	1.2825	225	0.38	1.7027	1.7168	0.8261	0.5584	0.5488	1.7205
1.053	0.003	0.025	1.2825	185	0.26	1.5043	1.4904	0.9235	0.4427	0.4371	1.2694
1.053	0.003	0.025	1.2825	185	0.34	1.4674	1.5214	3.6829	0.4262	0.4195	1.5736
1.096	0.003	0.025	1.2825	205	0.3	1.6501	1.6752	1.5216	0.5452	0.5594	2.6128
1.152	0.003	0.025	1.2825	245	0.26	2.1495	2.1115	1.7684	0.7729	0.7638	1.1805
1.179	0.003	0.025	1.2825	235	0.38	2.1645	2.1194	2.0831	0.7878	0.8138	3.2985
1.143	0.003	0.025	1.2825	155	0.34	1.6703	1.6098	3.6194	0.5861	0.5774	1.4918
1.072	0.0035	0.0225	0.3694	200	0.32	1.5347	1.5404	0.3742	0.5049	0.4935	2.2657
1.069	0.0045	0.0225	0.3694	200	0.32	1.5363	1.5076	1.8671	0.4521	0.4649	2.8243
1.071	0.0045	0.0325	2.8044	200	0.32	1.8086	1.7942	0.7938	0.6082	0.5887	3.1984
1.058	0.0055	0.0375	0.9781	200	0.32	1.8312	1.8044	1.4628	0.6325	0.6463	2.1864
1.067	0.0055	0.0275	0.9781	200	0.32	1.6125	1.6795	4.1559	0.6084	0.6134	0.8215
1.069	0.0065	0.0275	1.5869	200	0.32	1.6193	1.6534	2.1035	0.5678	0.5772	1.6478
1.065	0.0065	0.0325	2.1956	200	0.32	1.7578	1.7092	2.7642	0.5058	0.4932	2.4961

*V*: voltage at transformer bus before switching (p.u.), *R*_eqv_: equivalent resistance (p.u.), *L*_eqv_: equivalent inductance (p.u.), *C*_eqv_: equivalent capacitance (p.u.), L. L.: line length (km), *L*_sat⁡_: saturation curve slope (p.u.), error_*V*_: voltage error (%), and error_*T*_: duration time error (%).

**Table 3:** Case 1 some sample testing data and output.

*V*	L. L.	*V*_PSB_	*V*_RBFNN_	Error_*V*_	*T*_PSB_	*T*_RBFNN_	Error_*T*_
0.9541	150	1.3184	1.2898	2.1672	0.1067	0.1102	3.2816
0.9633	163	1.3675	1.3575	0.7314	0.2914	0.2951	1.2762
1.0272	178	1.4533	1.5037	3.4694	0.3806	0.3986	4.7228
1.0418	195	1.4867	1.4674	1.2951	0.4572	0.4463	2.3751
1.1069	207	1.6114	1.6891	4.8216	0.5153	0.5295	2.7495
1.1146	215	1.6602	1.7073	2.8357	0.5496	0.5684	3.4293
1.1862	230	1.8713	1.8391	1.7224	0.6132	0.6034	1.5907
1.2003	242	1.9741	1.9912	0.8639	0.6948	0.7147	2.8659

*V*: voltage at transformer bus before switching (p.u.), L. L.: line length (km), error_*V*_: voltage error (%), and error_*T*_: duration time error (%).

**Table 4:** Case 2 some sample testing data and output.

*V*	L. L.	*V*_PSB_	*V*_RBFNN_	Error_*V*_	*T*_PSB_	*T*_RBFNN_	Error_*T*_
0.9567	155	1.3012	1.3235	1.7135	0.0746	0.0767	2.8214
0.9669	170	1.3256	1.3173	0.6281	0.1472	0.1539	4.5184
1.0306	185	1.4219	1.3687	3.7428	0.3711	0.3617	2.5369
1.0435	200	1.4792	1.5025	1.5746	0.4259	0.4205	1.2657
1.1057	210	1.6025	1.6393	2.2974	0.4927	0.5034	2.1624
1.1204	225	1.6852	1.6328	3.1108	0.5483	0.5408	1.3751
1.1882	237	1.8395	1.8904	2.7659	0.6297	0.5984	4.9752
1.2033	250	1.9129	1.9412	1.4782	0.6736	0.6927	2.8395

*V*: voltage at transformer bus before switching (p.u.), L. L.: line length (km), error_*V*_: voltage error (%), and error_*T*_: duration time error (%).
